# Understanding Barriers to Surgical Care: A Qualitative Study of Black Individuals With Hand Dysfunction

**DOI:** 10.1111/hex.70958

**Published:** 2026-11-04

**Authors:** Noelle T. Thompson, Natalie B. Baxter, Ayana Cole‐Price, Mary E. Byrnes, Rachel C. Hooper

**Affiliations:** ^1^ University of Toledo College of Medicine and Life Sciences Toledo OH USA; ^2^ Center for Healthcare Outcomes and Policy University of Michigan Ann Arbor MI USA; ^3^ Division of Plastic Surgery The University of California Los Angeles CA USA; ^4^ Section of Plastic Surgery, Department of Surgery University of Michigan Ann Arbor MI USA; ^5^ Department of Surgery University of Michigan Ann Arbor MI USA

**Keywords:** community engagement, hand surgery, health disparities, health services research, patient‐centred care, qualitative research, surgical disparities

## Abstract

**Objective:**

To identify barriers influencing treatment decision‐making among Black individuals with carpal tunnel syndrome and to explore mechanisms contributing to disparities in hand surgery utilisation.

**Summary Background Data:**

Racial disparities exist in a variety of surgical subspecialties, including hand surgery. Little information on why these disparities exist or how to improve disparities from the patient's perspective is known.

**Methods:**

We conducted a study utilising virtual semi‐structured interviews, focusing on participants’ interactions with healthcare providers, the impact of hand dysfunction, and participants’ experiences with racism and discrimination. Using an interpretive description approach, we captured major themes in the coded data.

**Results:**

The sample included 28 Black individuals with carpal tunnel syndrome. We identified three key themes: (1) Racism predisposes Black participants to inequitable care: participants experienced intergenerational drag, microaggressions, discrimination, and mistrust related to historical injustices. Participants engaged in code‐switching to navigate biases and have their symptoms validated. (2) Social and systemic factors enable access to care: participants experienced economic instability, limited paid leave, unreliable transportation, inequitable workplace policies, and lack of social support. (3) Inadequate assessment of needs and access to care among Black participants: Participants struggled to navigate the referral process and obtain timely diagnostic testing. Many participants also described how providers misinterpreted their symptoms, which strained relationships with providers.

**Conclusions:**

Racism and discrimination contribute to Black individuals’ reluctance to undergo hand surgery. Improving disparities in surgery requires systemic and interpersonal solutions that optimise resource allocation, improve the surgical referral process, mitigate bias in healthcare interactions, and strengthen patient support networks. Understanding the drivers of racial disparities from patients’ perspectives is necessary to develop actionable, patient‐centred solutions.

**Patient or Public Contribution:**

Prior to study initiation, three structured community engagement sessions were conducted to collaborate with Black community members to co‐develop and iteratively shape our research question and study design. Our community research collaborators assessed study relevance, refined key study objectives, and informed interview guide domains.

## 1 Introduction

Racial disparities in access, treatment, and postoperative outcomes are well‐documented across multiple surgical subspecialties including general, colorectal, neurosurgery, cardiothoracic, orthopaedic, and vascular [1, 2, 3]. The mechanisms that drive racial disparities in surgical treatment are multifactorial and include access to timely referrals, social determinants of health, and patient willingness to undergo definitive surgical treatment. In a national survey‐based study, 22% of Black individuals reported that they avoid medical treatment, even when indicated for fear of discrimination [4]. Mistrust and differing expectations between the patient and provider may also explain the reluctance of Black individuals to choose surgical treatment [1, 5]

Carpal tunnel syndrome (CTS) is the most common compressive neuropathy of the upper extremity, resulting in numbness, tingling, nocturnal hand pain, and weakness. Treatment varies depending on severity and includes splinting, steroid injections, and carpal tunnel release [6, 7].

Despite the frequency of this diagnosis, racial disparities exist in access to surgical care and treatment decisions among Black patients with CTS. Delays in care can profoundly impact an individual's hand function, as well as their ability to perform personal care, enjoy hobbies, and earn wages. Black patients are less likely to undergo carpal tunnel release compared to their White counterparts; in one study that included 92,921 patients with CTS, Black patients were less likely to undergo surgery compared to White patients (19% vs. 44%, respectively, *p* < 0.001) [8]. Similarly, when comparing treatment choices among Medicaid patients, the authors found that a greater proportion of White patients compared to Black patients (21% vs. 14%, respectively) chose surgery as their first and only option [9].

Although racial disparities in the treatment of CTS and other surgeries have been described, it is unclear what drives these differences from the patients’ perspective. To understand the major contributors for racial disparities in surgery, we performed a qualitative study to examine the impact of lived experiences with racism and discrimination on treatment decisions among 28 Black participants with CTS. Given the consequences of undertreatment, understanding the systemic and structural factors that drive racial disparities is critical to identify strategies to mitigate these differences.

## 2 Methods

### 2.1 Study Design

For this study, we used interpretive description, a non‐categorical qualitative method that aims to understand applied clinical problems [10]. One‐on‐one semi‐structured interviews were conducted with 28 Black individuals about their hand health, experiences with racism and discrimination, as well as treatment decisions. IRB approval for this study (HUM00223362) was obtained from the University of Michigan Institutional Review Board.

### 2.2 Community Engagement

Prior to initiating the study, we conducted three community engagement sessions (virtual and in person) with a total of 24 Black individuals as a broader community engagement research approach to garner perspective and incorporate expertise from our targeted patient population. Community members served as research collaborators and were not interviewees. At the start of each community engagement session, background knowledge was shared regarding what carpal tunnel syndrome is, common symptoms of carpal tunnel syndrome, how carpal tunnel syndrome is diagnosed, and treatment options (splinting, steroid injections, and surgery) to establish a knowledge base and create a foundation for shared dialogue. Collaborators were asked to reflect on factors influencing their decision‐making to seek care, barriers to receiving care, perceptions of provider characteristics (race, sex, and age), prior experiences navigating hand conditions, and preferred formats for educational outreach. The research question, aims, and study design were also shared with collaborators. Collaborators reflected on study relevance and provided feedback on the research question and study design. Insights generated from these sessions directly influenced the research question and informed the development of the domains of our interview guide.

### 2.3 Participant Recruitment and Selection

Participant inclusion criteria were the following: (1) self‐identification as Black, (2) > 18 years old, (3) English‐speaking, and (4) formally diagnosed with CTS. We used purposive sampling through multiple recruitment avenues to identify Black adults with hand dysfunction who could provide perspectives relevant to the study aims. We recruited participants from a single academic institution in the Midwest who presented to an outpatient hand surgery clinic. We also recruited participants through the Michigan Institute for Clinical and Health Research participant recruitment programme, which uses social media to advertise clinical studies to the local community [11]. This programme facilitates access to participants outside of our immediate health system with specific medical diagnoses through linkage with their electronic health records. Interviews were conducted between October 2023 and March 2024. As such, participants have heterogeneous healthcare experiences in terms of access to public or private healthcare systems and surgical specialists. At the academic institution, patients with CTS are typically referred to a hand surgery specialist following a clinical diagnosis or after electrodiagnostic testing. All patients recruited from the hand surgery clinic were insured.

### 2.4 Interviews and Guide

The interview guide (NTT, RCH, and MEB) was developed through principles of interpretive description and informed by principles from the Andersen Access to Care Model [12] and the Public Health Critical Race Praxis using anti‐racism principles [13, 14]. The guide was structured to investigate (1) the personal, social and economic impacts of hand dysfunction, (2) the perceptions of quality of care and patient‐physician communication, and (3) the impact of racism and discrimination on treatment decisions for CTS. Interviews were conducted on a video‐based platform, recorded on an encrypted device, transcribed using a HIPAA‐approved AI transcriber, and manually cross‐referenced for consistency. NTT obtained consent from participants before conducting the interviews. All participants were interviewed using this interview guide and asked the same primary questions. Interviews generally lasted between 30 and 40 min, with the overall duration being between approximately 25 and 70 min. Consistent with a qualitative semi‐structured interview approach, NTT asked follow‐up questions to further explore narratives and clarify perspectives that participants shared. Interview duration therefore varied between participant communication styles and the need for in‐depth probing. Some participants conveyed substantive and valuable perspectives in a more concise manner. All interview transcripts were imported into MaxQDA [15], a qualitative data analysis software, for evaluation. Each participant received a $50 check for remuneration for their time.

### 2.5 Analysis

Members of the team (NTT, RCH, MEB, and NBB) individually read through transcripts to identify codes present in participant narratives. The team met weekly to discuss similarities and differences between codes until consensus was reached for a working iterative codebook. The codebook was then tested on three interviews to ensure it captured the participants’ voices while addressing our research question. Iterative modifications were made as new codes arose. The research team met to discuss patterns in the data and interpret the meaning. The team wrote three iterations of theme reports as discussion evolved.

## 3 Results

Twenty‐eight individuals participated in the study. The majority were women (82%), younger than 50 years old (57%), and had occupations that involved manual labour (39%) Supplementary Table.

### 3.1 Theme 1: Racism Predisposes Black Participants to Inequitable Care (Table 1)

**Table 1:** Racism predisposes Black participants to inequitable care.

Theme	Definition	Participant	Exemplary
Racism predisposes Black participants to inequitable care			
	Expressed experiences of interpersonal or systemic racism and how that impacts their view of the healthcare system, their ability to access care, or health outcomes	10	‘I personally feel if I was a white, young woman, same age, [age number], that my care would have been a hell of a lot different than it was both in the hospital when I was in there for two months.’
		20	‘I felt like if I was of a different race that it would have gone smoother. I felt like I was being rushed out of there from the time that I arrived to the time that it was over.’
		9	‘Black people are scared to go under the knife. We just are. I think it has a lot to do with that situation with my child where it's like, wow, really? Am I going to get the best care while I'm under?’
		26	‘In fact, I have to say that we selected the [hospital] because I was under the impression that it was the best. And I have been a bit underwhelmed at the lack of diversity or the inability to access providers of colour or, you know, just the, you know, the lack of communication.’
	Expressed how intergenerational drag impacts their decision‐making on seeking care	15	‘I believe that some African Americans, they got in their head that they're not going to get the best care when they go, that they're going to get mediocre care. They are going to be pushed to the side or everybody else going to be more important than them.’
		1	‘A lot of time in the Black community, we wait until the very last minute to get stuff done or don't do it at all. A lot of times, there's the fear of how you'll be treated, how you'll be looked at, how you'll be perceived. A lot of times, it's generational.’
	Expressed a lack of quality local care in Black neighbourhoods and how that impacts care	12	‘I've gotten the worst treatment where the areas of concentration are brown skin but gotten the best treatment when I leave outside my health system to go where they're in the habit of doing the best based on that socioeconomic level of that area.’
		23	‘The locations, the quality of health services, or the lack thereof for anyone is transportation. So those without transportation are just left to go to all of these clinics that can care less about their health.’

Participants in our study reported experiencing race‐based discrimination during previous clinician encounters prior to evaluation by a hand surgeon for CTS.

#### 3.1.1 Intergenerational Drag and Systemic Racism Contribute to Medical Mistrust

Previous exposure to inequities in healthcare influenced participants’ perceptions about CTS treatment. Many described intergenerational drag, or the concept that the effect of past discrimination is cumulatively passed down across generations and influences present health behaviours [16]. For several participants, knowledge of prior traumatic events leads to feelings of mistrust of surgery as a type of ‘experiment’. This participant explained their thoughts when they were offered surgical treatment for their hand condition:

They still talk about the Tuskegee thing…I do feel like African Americans get less assertive treatment…We do have a negative history with medical experiments. And so, I know going under the knife is a frightening prospect for anyone. But for Black people…it does make you afraid. We don't want to be used as experiment rats.

(P13)

Some participants explained that generational mistrust has led members of the Black community to avoid seeking medical care altogether, perceiving it as a protective response against exploitation [17, 18]. Participant 15 explained:

Sometimes Black people feel like they are test tubes. They're the ones they use to test on, make other races better. And then it's a lot of things that happen to them in the early years that stopped them from going to the doctor.

(P15)

CTS is the most treated hand surgery condition, with high success rate and low incidence of complications [19]. Despite this, some participants experienced macro and micro forms of racism; these encounters undermined their trust in providers and led them to believe that quality care was not accessible. One participant shared:

I know you have some of the best doctors in the world, but if they don't treat me, then it doesn't make any difference to me if I don't receive that world‐class treatment because of the colour of my skin.

(P2)

Their experiences indicated that the delivery of ‘*world‐class treatment’* by large academic hospitals is overshadowed by care that is discriminatory, racist, or lacking in cultural humility. Furthermore, participants shared their perception that Black individuals are not valued in the same way other races are and are treated as ‘less than’ by healthcare professionals. As such, many of our participants felt that their CTS treatment would be discriminatory and substandard in comparison to the treatment that other racial groups receive. Ultimately, this led them to avoid the medical system (Figure 1).

**Figure 1:**
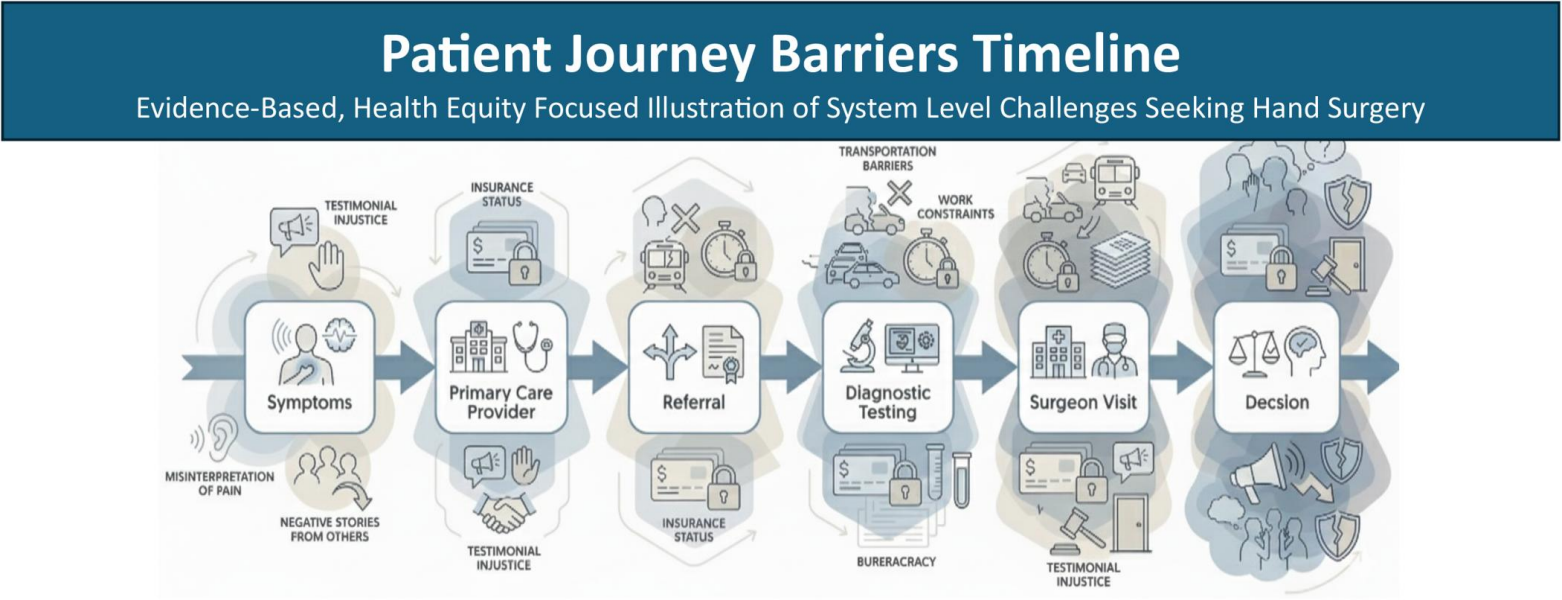
Patient Journey For Carpal Tunnel Syndrome Care. Illustration of the patient journey from symptom recognition through primary care evaluation, referral, diagnostic testing, surgical consultation, and treatment decision‐making. At each stage, patients may encounter interpersonal, structural, and systemic barriers, including misinterpretation of pain, testimonial injustice, insurance limitations, transportation difficulties, work constraints, and administrative bureaucracy. These barriers may compound throughout the care continuum, influencing healthcare experiences, access to timely treatment, patient perspective and surgical decision‐making.

#### 3.1.2 Lack of Quality Local Care

Participants shared how structural inequity in access to nearby care led to delays in presentation to a hand specialist and preference for nonsurgical treatment. Participant 23 explained:

One of the things that I'm fighting with in my community is the health disparities…the quality health services, or the lack thereof…those without transportation are just left to go to all these clinics that can care less about their health.

(P23)

Insufficient high‐quality healthcare services in their predominantly Black neighbourhoods [20, 21] compounded with transportation issues predisposed participants to suboptimal outcomes. Individuals without transportation [22, 23] attended local clinics with less expertise and resources to treat their conditions. Others struggled to reach specialty care further away from home. Individuals shared details about their ‘fight’ to overcome the underinvestment in quality healthcare services in their local communities and highlighted the need for increased screening for social determinants of health, including transportation services, as a strategy to address this disparity [24, 25].

### 3.2 Theme 2: Social and Systemic Factors Enable Access to Care (Table 2)

**Table 2:** Social and systemic factors enable access to care.

Theme	Definition	Participant	Exemplary
Social and systemic factors enable access to care			
	Described how their social networks and the inherent challenges of their environment limit their ability to seek care for CTS, contributing to disparities in treatment decisions and outcomes	3	‘My job tried to fire me for the diagnosis. So, when I would go to my boss and complain about the pain in my hand, he would just send me home…He didn't want me to go into the doctor…I got diagnosed for the second time in November of 2022. I didn't have my surgery until May 11th 2023. That's how long it got dragged out. I was in pain every day.’
		18	‘I got [the surgery for CTS], when I was working at the bank… I don't know how to explain that other than they thought you just wanted time off work if you had CTS. And as a Black person, a lot of times you end up feeling challenged, like, I'm not trying to fake. So here, let me show you. You know what I mean? I won't take time off because everybody thinks you want time off.’
		26	‘I think it's an issue of just access gateway. If you don't get in the first door, you're not going to get in the fourth door. So if you don't have a primary care, who then refers you then to a specialist, who then is willing to follow the science and follow best medical practice in terms of your treatment, or ‘she's fine.’ She's just trying to get off work for six weeks, or it's all the things that they impute to people of colour who are accessing or trying to access care.’
		7	‘Probably medical insurance for most and wanting to just I know as black men, most of the time we tend to put our ailments to the side because we either don't want to miss out on money or don't want to miss work.’
		22	Scared [to get CTS surgery] because I don't know how I'm going to be able to take care of myself after while I'm healing. Who is going to take care of me?’

Participants described how their social networks, work policies, and insurance structures affected their ability to seek care for CTS, contributing to disparities in treatment options.

#### 3.2.1 Lack of Employer Support

Many participants worked in manual labour roles and described how employer support or lack thereof caused delay in care for CTS. This participant recalled:

My doctors just felt like I was lazy, trying to get something out of the deal, workers comp or either disability or something, but no one would really take it serious for a long time. …It was hard for me to actually get the real test because they just thought I was faking…maybe because I was a big guy and athletic…

(P16)

As described here, Participant 16 shared that the racially charged laziness trope [26, 27] was weaponized against him by those he sought medical care from.

#### 3.2.2 Insurance Coverage Influences Treatment Options

Participants described how existing insurance structures [28, 29], shaped by systemic racism [30], perpetuate disparities and inequitable treatment:

And I had good insurance, so that played a role, too, because now my having to go out of work due to disability and the insurances in which I received, I can see that I don't get the same attention that I did when I had what they consider credible insurance versus, I guess it's government insurance, Medicare.

(P23)

This participant perceived that the quality of care they received was influenced by their insurance status. They suggested that lacking credible insurance may have limited their access to comprehensive evaluations and full disclosure of treatment options. In other instances, underinsurance coupled with financial constraints contributed to a fear of seeking surgical treatment for CTS, and ultimately led to avoidance of surgery. Participant 18 explained:

I was scared to do the surgery. A lot of it was economic, because I'm on disability now. But it's only been since then that I've had regular health insurance. And even then, it was often prohibitive as far as the time taken off for the surgery or the cost for the surgery.

(P18)

As participant 18 explained, their fear of surgery was intertwined with their economic concerns. Other participants shared the sense that some surgeons were disingenuous and financially driven when offering certain treatments (Table 2). Participants who held private insurance shared a sense of security and autonomy as this asset allowed access to multiple providers and healthcare systems. Although many participants implored healthcare professionals to treat all CTS patients equitably regardless of insurance status, they also emphasised how private insurance served as protection against racism and as a tool to increase access to quality care.

### 3.3 Theme 3: Inadequate Assessment of Needs and Access to Care Among Black Patients (Table 3)

**Table 3:** Inadequate assessment of needs and access to care among Black patients.

Theme	Definition	Participant	Exemplary
Inadequate assessment of needs and access to care among Black patients			
Symptom Validation	Expressed discrepancy between the severity of their symptoms and the interpretation of their symptoms by health care providers	4	‘So that was my fear [of getting the surgery], I think, was that I was going to be ignored about my pain. And that was something that I've heard throughout the years of people being in pain and people being ignored, but I think that was my biggest fear.’
		22	‘I got tired of trying to convince doctors that I'm hurting bad enough to get stronger medication, to get different medication, and then the backlashes. I get treated like I'm a drug addict.
		23	‘Because there are racial disparities all around us, and that's just a part of what's going on. I don't know…I hadn't been actively involved in my own health care, I may have never been referred to a hand specialist.’
Differing thresholds for surgery	Expressed varying thresholds for undergoing surgery based on hand dominance and severity of hand pain	15	‘No, but I kind of was a little hesitant…I am thinking [the surgery for CTS] is supposed to stop it. But after my own pain and understanding that some less pain will be better than more pain.’
		11	‘So I can work around that because I'm a right hander. But doing the right hand, I really needed to make sure that it wasn't going to interfere with anything that with school and stuff like that. So that's the only reason I really waited so long.’
		13	‘Well, first of all, it's my right hand and that's my dominant hand. I was really apprehensive because even having the surgery, I know when you have surgery, things are just not ever quite the same. And I think my fear was having the surgery and then the situation be worse as opposed to better.’
Lack of knowledge regarding CTS	Expressed knowledge gaps regarding CTS treatment	27	‘There were no [treatment options shared with me]. And after that appointment, I really didn't go back and seek any more treatment because I just didn't like how the visit went.’
		28	‘And I think even with African Americans and counterparts, a lot of it just have to be lack of knowledge, what's going on. And it's kind of hard to make an educated decision about something when you really don't know anything about it.’

#### 3.3.1 Symptom Validation

Many of our participants described a discrepancy between the severity of their symptoms and how health care providers interpreted them. Individuals shared how their outward expressions of pain (i.e., facial expressions or tears), or lack thereof, often led to misinterpretation [31, 32, 33, 34]. This discrepancy between symptom presentation and interpretation led to testimonial injustice [35]. Participants felt that their experiences of severe pain and concerns with their hands were not addressed as they would be for non‐Black individuals:

Doctors think that we're not really in pain, and we're not used to expressing that this really hurts. We will say, like, ‘it hurts.’ And they'll be like, ‘oh, well.’ That's because I'm not crying, and all of that.

(P2)

Likewise, participants shared how the different interpretation of body expressions led to inaccurate documentation of pain that was perpetuated in the medical record, contributing to ongoing racial disparities in care. For some, the lack of symptom validation was increasingly frustrating and placed additional strains on the patient–provider relationship:

I think I can tolerate a lot of pain; I think. And I just wasn't crying and acting like a big baby, but I was in pain, but I just kept doing what I had to do.

(P16)

### 3.4 Differing Thresholds for Surgery

We found that hand dominance and severity of hand pain were major drivers in the decision to have surgery. Participants were less likely to undergo surgery for CTS when their dominant hand was involved. In contrast, those with severe pain were more willing to undergo surgery. One participant shared:

I really needed to make sure that it wasn't going to interfere with school and stuff like that…that's the only reason I…waited so long.

(P11)

The decision for surgical treatment was predicated on the ability to continue with work and other activities of daily living. It was more important to work with impairments and mild pain, than to take on the risks of surgery.

Additionally, our data demonstrated that negative experiences with CTS treatment among friends, family, and co‐workers impacted participants’ treatment decisions:

People from work. That was the other thing that discouraged me was they seemed to act like getting the surgery wasn't like a help to them.

(P18)

The risk of incomplete relief of symptoms or other complications following surgery made participants reluctant to pursue this form of treatment.

## 4 Discussion

Prior experiences with racism and discrimination when interfacing with the healthcare system impact treatment decisions among Black patients seeking CTS care. Black patients are less likely to undergo surgery for CTS and when they do, it is more likely to occur more than 6 months following their diagnosis; this is a significant difference compared to their White counterparts [9]. Although previous studies have identified these disparities in hand surgery and across other surgical subspecialties, little has been done to understand why they occur from the patient's perspective [8, 9]. In this qualitative study, we found that (1) racism predisposes Black participants to inequitable care 2) social and systemic factors enable access to care and (3) inadequate assessment of needs and access to care impact treatment decisions and quality of care. Beyond CTS, Best et al. [1] identified disparities between Black and White patients in the utilisation of several surgical procedures, including spinal fusion, coronary artery bypass, total knee, and hip replacement [1]. Based on our results, this may be partially explained by the perceived interpersonal and structural racism that unfavourably affects the ability of certain individuals to access care. When participants in our study experienced racism, this led to avoidance of the medical system and reluctance to consider definitive surgical treatment. Based on our participants' shared experiences, physicians may consider incorporating the following questions: (1) Is there anything that we should be aware of from your prior experiences with the healthcare system that negatively impacts your medical decisions, as well as how and when you access care? (2) If so, what can we do to make you more comfortable? Furthermore, many large academic institutions and state medical licensing bureaus require physicians to complete online training on bias mitigation and strategies for providing culturally competent care. Given our findings, perhaps there is a role for attending in‐person sessions and participation in race‐ or culture‐specific hypothetical scenarios to improve this aspect of care.

Insufficient personal and societal support networks among Black participants limits their ability to pursue certain treatment options and renders them less likely to seek surgical care. Economic instability, lack of paid leave, unreliable transportation, inadequate insurance, and lack of social networks impacted participants’ treatment decisions. Previous studies have demonstrated that Black patients are more likely to undergo steroid injections, which typically provide immediate but short‐term relief from wrist pain. In addition, among those on Medicare, Black patients have a higher likelihood of choosing steroid injections over surgery for treatment [8, 34]. For patients with moderate‐to‐severe disease, delay in surgery can result in suboptimal outcomes such as permanent hand numbness, weakness, and overall dysfunction [36, 37, 38, 39, 40]. Despite these devastating consequences, participants in this study described a tendency to favour steroid injections because of their simplicity, non‐invasive approach, and the immediate ability to continue work and other activities. Inequitable assessment of needs and access to hand surgeons contributes to disparities in the treatment of Black patients. Participants in our study who lacked primary care providers struggled to obtain a diagnosis, while others were confused about the need for referral to a neurologist versus a hand surgeon. Inconsistencies in symptom validation among Black patients have been demonstrated in several studies, particularly under circumstances of patient‐provider racial discordance [41, 42, 43, 44, 45]. In one study, many critically ill Black patients reported epistemic injustice, or the silencing of their knowledge and lived experiences by healthcare providers; this exacerbated mistrust and led to poor outcomes [41]. Similarly, we found that symptom validation is crucial for patients to obtain the appropriate referrals [46], diagnostic tests [47, 48], and definitive surgical treatment. Our findings suggest that an increased emphasis on understanding the lived experiences of Black patients [49], treatment expectations, and differences in the manifestation of pain [50] can help minimise medical mistrust, improving access and quality of care for Black patients. Based on our participants' recommendations (Table 4), we propose the following questions be incorporated in the preoperative patient encounter: (1) How is your condition impacting your everyday life? 2) What resources do you have readily available to you to be able to access the treatment options that we discussed today? 3)What are your expectations regarding the treatment process and recovery? 4) What concerns, if any, do you have regarding the treatment options discussed today? and (5) How can our team make sure that you feel respected and heard in your care? With the addition of these questions, providers can gain a more holistic view of the many drivers that impact patient surgical treatment decisions.

**Table 4:** Proposed solutions to improve disparities in hand surgery.

Solution	Participant	Exemplary
Understand how the condition impacts quality of life	16	‘And I'm just trying to get fixed so I can continue to work. That's the main issue. Everybody's not lazy. Everybody don't want pills…They should listen to a person and evaluate a person to see what's going on before they start making decisions.’
	20	‘I would want [surgeons] to know that I use my hands to work, and I'm very careful and very cautious with my work ethic and my lifestyle and with just everything I do.’
	24	‘And again, I would say that for any doctor, for any. Listen to the patient, find out what they do every day. See how that, like you've been asking me the questions. I think that is something that a lot of doctors, especially surgeons, sometimes they just [say], ‘Well, your doctor wanted me to look at you.’’
	26	‘It's critical that if [the surgeon has] the ability to help or improve or correct something that has become a significant issue, significant enough that the person is describing it affecting their ability to work, to parent, to study, to be a student, to whatever, that it's even more important that they provide what they can to assist because they have the ability to influence the person's trajectory.’
Identify expectations and goals that the patient has regarding the treatment and recovery process	18	‘I guess part of it would be [surgeons] learning the right questions to ask and understanding that their lifestyle might be different than yours. Like, they'll say things like that. ‘We'll just have someone come in and help you for a few days.’ A lot of times I think they mirror what their lives are, and they think that you have the same opportunities in lifestyle.’
	1	‘Communicate. Please communicate with the patient. Let the patient know every step of the way what you're doing, what your role is, why they're here, even if you might think that they know why they're there. A lot of times people might not necessarily know what to expect from that experience.’
	5	‘I think the only thing that I wanted to know a little bit better was the recovery was more painful than they let on. I think because it's a routine surgery, they just explained it like, it was really simple…and just your mobility and stuff were more limited than I thought it was going to be… And so I think if that was explained to me better, I would have had more realistic expectations of how soon I would have went back to work.’
Examine patient's concerns and acknowledge them	11	‘Make them feel like what they're saying is important. And I don't even think it just falls on surgeons in general, like doctors as a whole, be aware of the stigma that the Black community has when it comes to the healthcare.’
	4	‘So that was my fear, I think, was that I was going to be ignored about my pain. And that was something that I've heard throughout the years of people being in pain and people being ignored, but I think that was my biggest fear. But they seemed to be receptive to the fact that I was in pain.’

## 5 Limitations

This study has several limitations. First, our sample was drawn from a single academic institution, which may not fully capture the breadth of Black individuals’ experiences with CTS, particularly those from different regions or with limited social media access. Second, while our qualitative study offers rich insights into patient experiences, it is inherently subjective, influenced by participant perspectives and researcher interpretations. Participants may have underreported or overemphasised aspects due to recall bias, social desirability, or perceived expectations. We employed rigorous coding strategies and iterative analysis to enhance credibility and reflexivity; however, researcher positionality may have influenced interpretation. Moreover, generalisability is constrained by the inclusion of only English‐speaking participants and a predominantly younger, female demographic. Future research incorporating provider interviews and institutional analyses may offer insights into structural contributors to disparities and inform interventions. Despite these limitations, this study provides valuable insights into Black individuals’ experiences with CTS and highlights critical areas for improving equitable access to care and surgical decision‐making.

## 6 Conclusion

We found that experiences with racism and discrimination lead to medical mistrust, delay in presentation, and avoidance of definitive surgical treatment among Black individuals with CTS. Participants felt dismissed by healthcare providers, misunderstood in their expressions of pain, and subject to biased assumptions about their symptoms and work ethic. Strategies to avoid the use of language and behaviour that can be perceived as racist or discriminatory, along with validation of patients’ experiences and symptoms, can help narrow the racial disparities and improve the quality of care for individuals with this common condition.

## Author Contributions

**Noelle T. Thompson:** writing – original draft, writing – review and editing, formal analysis, data curation. **Natalie B. Baxter:** data curation, writing – review and editing. **Ayana Cole‐Price:** data curation, writing – review and editing. **Mary E. Byrnes:** writing – original draft, writing – review and editing, methodology, validation, data curation. **Rachel C. Hooper:** conceptualisation, investigation, writing – original draft, writing – review and editing, methodology, formal analysis, supervision, data curation.

## Funding

The authors have nothing to report.

## Ethics Statement

This study was conducted in accordance with the Declaration of Helsinki and approved by the Institutional Review Board (IRB) of at the University of Michigan, HUM00223362. Written/oral informed consent was obtained from all individual participants prior to their inclusion in the study. Special care was taken to ensure the confidentiality and anonymity of all public and patient contributors.

## Conflicts of Interest

The authors declare no conflicts of interest.

## Supporting information


Supporting File 1

## Data Availability

The data that support the findings of this study are available on request from the corresponding author. The data are not publicly available due to privacy or ethical restrictions. Rachel Hooper had full access to all the data in the study and takes responsibility for the integrity of the data and the accuracy of the data analysis. Deidentified participant data are available upon request to hooperra@med.umich.edu.
