# Supplementary material for: Understanding Barriers to Surgical Care: A Qualitative Study of Black Individuals With Hand Dysfunction
Source: Health Expect. 2026 Nov 4;29(6):e70958. doi: 10.1111/hex.70958 (PMC13732644; doi:10.1111/hex.70958)
Supplement: Supplementary file 1 [file HEX-29-e70958-s001.docx]

**Participant Characteristics**

**Table 1. Summary of Participant Demographic Characteristics (N = 28)**

| **Characteristic** | **Participants** |
| --- | --- |
| Age range, years | 26–74 |
| **Gender** |  |
| Female | 23 (82.1%) |
| Male | 5 (17.9%) |
| **Occupation** |  |
| Manual labor | 11 (39.3%) |
| Mixed | 9 (32.1%) |
| Healthcare | 3 (10.7%) |
| Education | 2 (7.1%) |
| Unemployed | 2 (7.1%) |
| Retired | 1 (3.6%) |
| **Race** |  |
| Black/African American | 28 (100%) |

**Table 2. Individual Participant Characteristics**

| **Participant** | **Age, y** | **Race** | **Occupation** | **Gender** |
| --- | --- | --- | --- | --- |
| P01 | Not Reported | Black/African American | Healthcare | Female |
| P02 | Not Reported | Black/African American | Mixed | Female |
| P03 | 28 | Black/African American | Manual Labor | Female |
| P04 | 35 | Black/African American | Mixed | Female |
| P05 | 38 | Black/African American | Mixed | Female |
| P06 | 40 | Black/African American | Manual Labor | Female |
| P07 | 32 | Black/African American | Manual Labor | Male |
| P08 | 46 | Black/African American | Manual Labor | Male |
| P09 | 45 | Black/African American | Manual Labor | Female |
| P10 | 36 | Black/African American | Mixed | Female |
| P11 | 33 | Black/African American | Manual Labor | Male |
| P12 | 58 | Black/African American | Mixed | Female |
| P13 | 69 | Black/African American | Mixed | Female |
| P14 | 26 | Black/African American | Unemployed | Female |
| P15 | 57 | Black/African American | Manual Labor | Female |
| P16 | 59 | Black/African American | Manual Labor | Male |
| P17 | 49 | Black/African American | Manual Labor | Female |
| P18 | 57 | Black/African American | Mixed | Female |
| P19 | 36 | Black/African American | Manual Labor | Female |
| P20 | 31 | Black/African American | Mixed | Female |
| P21 | 67 | Black/African American | Manual Labor | Female |
| P22 | 50 | Black/African American | Unemployed | Female |
| P23 | 60 | Black/African American | Education | Female |
| P24 | 74 | Black/African American | Healthcare | Female |
| P25 | 37 | Black/African American | Education | Female |
| P26 | 72 | Black/African American | Retired | Female |
| P27 | 46 | Black/African American | Healthcare | Female |
| P28 | 60 | Black/African American | Mixed | Male |
